# Love and Suicide: The Structure of the Affective Intensity Rating Scale (AIRS) and Its Relation to Suicidal Behavior

**DOI:** 10.1371/journal.pone.0044069

**Published:** 2012-08-29

**Authors:** Zimri S. Yaseen, Karin Fisher, Esperanza Morales, Igor I. Galynker

**Affiliations:** Department of Psychiatry and Behavioral Sciences, Beth Israel Medical Center, New York, New York, United States of America; University of Pennsylvania, United States of America

## Abstract

**Background:**

Suicide has been linked to intense negative affect. However, little is known about the range of affects experienced by suicidal persons, or the separate effects of affect valence and intensity. We examine a novel self-report scale, the 17-item Affective Intensity Rating Scale (AIRS), and its relation to suicidality in a high-risk sample.

**Methodology/Principal Findings:**

Patients presenting with suicidality were recruited from the Emergency Department in a large urban hospital, and completed a battery of assessments there. Structure of the AIRS was assessed using Maximum Likelihood Factor Analysis with Oblimin rotation. Convergent and divergent validity were assessed by regressing AIRS subscales against Brief Symptom Inventory subscales. Relation to suicidality was assessed by regression of suicide attempt status against scale and subscale scores, and individual items and two-way item interactions, along with significant clinical and demographic factors. 176 subjects were included in analyses. Three reliable subscales were identified within the AIRS measure: *positive feelings towards self, negative feelings towards self,* and *negative feelings towards other.* Only individual AIRS items associated significantly with suicide attempt status; strong ‘feelings of love’ associated positively with actual suicide attempt, while ‘feelings of calm’ and ‘positive feelings towards self’ associated negatively. Interaction analyses suggest ‘calm’ moderates the association of ‘love’ with suicide attempt.

**Conclusions/Significance:**

Factor analysis of the AIRS is consistent with a circumplex model of affect. Affective dimensions did not predict suicidal behavior, but intense feelings of love, particularly in the absence of protective feelings of calm or positive self-view associated with current attempt.

## Introduction

Suicide is a leading cause of mortality for in the United States more than 36,000 die by suicide annually, and nearly twenty times as many present to emergency rooms with suicide attempts [1], [2]. However, while chronic risk factors for suicide are increasingly well understood, we remain unable to predict acute suicide risk [3].

Recent research has thus begun to focus on acute states which may be markers of a “suicide-crisis.” [4] Suicide has been linked to states of high intensity negative affect [5], and prior to suicide death, patients experienced multiple intense negative affects in association with depression [6] **.** Affects such as hopelessness, rage, guilt, abandonment, loneliness, severe anxiety, humiliation, and self-hatred have shown to play an important role in suicidality [4]. Likewise, previous research suggests that the transition from suicidal ideation (SI) to suicide attempt (SA) may be triggered by states of high intensity negative affect [5],[7].

The distinct associations of affective intensity and affective valence with suicide have not been explored, however. Circumplex models of affect describe affects in terms of independent components such as valence and arousal [8], [9]. While arousal can be understood as the degree to which a given affect associates with motivation for activity, individual affects may also vary in intensity [10]. While self-report scales such as the Emotional Intensity Scale (EIS) [11] and the Affective Intensity Measure (AIM) [12]–[14], have been developed to measure *trait* affective intensity, to date no study has examined the distinct contributions of *state* affective intensity and valence to suicidal behavior.

Further, another important dimension in affective experience, which is not assessed in the AIM or EIS, is directedness, i.e., is the affect aimed at the self or others. [15] Indeed, directedness of affect is linked to motivational states and influences behavioral response selection. [15] As suicide may be seen as an act of aggression towards the self and/or a communicative act expressing anger or despair to others, one might expect not only the valence of affects experienced, but also their self- or other-directedness to impact suicidal behavior. Indeed Bolton, et al., found that specific depressive symptoms of guilt and worthlessness – self-directed negative feelings – more so than sadness associated with future suicide attempt in a large population based study. [16].

Finally, little is known about the range and variety of affects experienced by suicidal persons. Though prior research suggests significant inverse association between suicidal ideation and positive affect [17], the role of individual affects, such as love, in suicidal behavior has received little quantitative attention [18]. Thus, it remains an open question whether general sectors of the affective spectrum (e.g. intense, negative, self-directed affect) or specific individual affects or affect combinations are more closely linked suicide.

On the one hand it might be expected that sectors of the affective spectrum such as intense, negative, self-directed affect, (as might be represented by a subscale of the AIRS), should capture a depressive process that leads to suicidality. On the other hand, though most suicide attempts are made by persons suffering depression at the time [19]–[21] the converse does not hold and suicide is, in absolute terms, fairly rare among depressed persons [20]. Thus, one might expect that suicide is driven not by the general depressive sector of the affective spectrum but by more specific individual affects or affect combinations.

To begin to answer these questions, we examined the structure of a novel scale, the 17-item Affective Intensity Rating Scale (AIRS), and its relation to suicidality in a psychiatric emergency room setting. This scale was designed to survey the degree to which a wide and balanced range of positive and negative affects were experienced intensely during the 72 hours leading up to psychiatric contact in the emergency room. In addition, the scale included four summary items examining subjects’ experience of the self- or other- directedness of their affects. (See Appendix S1 for complete scale.) We hypothesized that:

1. Factor analysis of the AIRS would identify subscales consistent with a circumplex model of affect comprising valence and directedness axes, thus generating “positive self”, “negative self”, “positive other”, and “negative other” subscales.
2. Greater affective intensity as measured by AIRS total score will associate with subjects’ presenting to the ER with current attempt, versus ideation only.
3. High intensity self-directed negative affects (“negative self”) will associate with current attempt, versus ideation only.
4. The AIRS will identify individual affects linked to current attempt, versus ideation only.

## Methods

### Design and Procedures

Data for this study was collected at Beth Israel Medical Center in New York City in conjunction with an emergency-room setting validation study of the Suicide Trigger Scale (Yaseen et al., 2010). The Beth Israel Medical Center Institutional Review Board approved the study. In this purposive non-probability sampling design, participants were identified and referred to the study by an emergency room clinician when suicidality was identified as part of the history of their present illness. During initial assessment, participants provided written informed consent. All data were analyzed anonymously and steps were taken to protect subjects’ identities; subject data were entered into a database for analysis in a de-identified manner. Eligible subjects who consented to participation in the study then received an in-depth assessment of suicide behavior by a trained research assistant during their stay in the emergency room. In addition, subjects who required significant medical care due to their suicide attempts were seen by the emergency room psychiatrists, and once medically stabilized, if eligible and consenting, were interviewed within 24 hours of their stabilization in the hospital. Measurements were administered in the same order to all participants. In addition, assessments were corroborated by comparison with the emergency room chart for each subject. Participants were reimbursed $25.

### Participants

Inclusion criteria were age between 21 and 65 years old, able to understand the consent form in English, and presentation with suicide ideation or attempt to the psychiatric emergency room. Exclusion criteria were mental retardation, cognitive impairment, or linguistic limitation precluding understanding of the consent process, possible delirium, or significant neurological disease, and prior or current diagnosis of malingering as assessed by the psychiatrist(s) in the psychiatric emergency room.

### Measures

#### Demographic variables

A demographic questionnaire was created for this study to elicit information on age, sex, marital status, level of education and household income.

#### Diagnosis

Diagnosis was assessed during clinical evaluation interview in the emergency room setting by a psychiatrist. Psychiatrists were experienced board licensed psychiatric emergency room staff psychiatrists or second and fourth year psychiatry residents directly supervised by the former. This data was then extracted from review of patient charts from the psychiatric emergency room, and was coded as 1) No DSM Axis I diagnosis, 2) Anxiety or unipolar depressive disorder without psychosis, 3) Bipolar disorder without psychosis, and 4) Psychotic disorder to maximize diagnostic reliability [22]–[24] as well as degrees of freedom, thereby increasing statistical power in subsequent analyses.

#### Substance use

Substance use was assessed during intake interview in the emergency room setting by a psychiatrist. This data was then extracted from review of patient charts from the psychiatric emergency room, and was coded as present if alcohol or drug abuse or dependence were reported.

### Assessment of Affective Intensity

#### Affective intensity rating scale

To rate and score the presence of high intensity in a wide range of emotional states in the 3 days preceding suicidal presentation in the psychiatric emergency room, we utilized a novel 17-item measure called the Affective Intensity Rating Scale (AIRS). The AIRS is a self-report measure. Patients were asked to rate whether their experience of each of several of mood states during the 72 leading up to their emergency room presentation was “unusually intense or deep” utilizing a 3-point Likert scale (0 =  not at all, 1 =  somewhat, 2 =  a lot) responses. Sample items assessing individual affects are presented below:

During the last 3 days before you came to the Emergency Room did you experience any periods where you felt the following:

Unusually intense or deep feelings of sadness?

0 =  Not at all. 1 =  Sometimes. 2 = A lot.

Unusually intense or deep feelings of love?

0 =  Not at all. 1 =  Sometimes. 2 = A lot.

A sample summary item used to assess self- versus other- directedness of positive and negative affects is presented below:

Any unusually intense or deep negative feelings directed towards yourself?

0 =  Not at all. 1 =  Sometimes. 2 =  A lot.

### Symptomatic Assessment

Symptomatic assessment of study participants was performed with the Brief Symptom Inventory (BSI). The BSI is a 53 item self-report instrument with nine symptom-domain subscales and a global severity subscale. [25].

### Assessment of Suicidal Behavior

#### Columbia suicide-severity rating scale

The C-SSRS [26] is a clinician-administered, semi-structured interview for the identification and standardized assessment of suicidal ideation and behavior in any setting. The presence of suicidal ideation was ascertained at five levels of increasing degrees of ideation severities. These included passive ideation (wish to be dead or go to sleep and not wake up) and four levels of active ideation (thoughts of killing self, thoughts of method for killing self, intentions to kill self, and development of plans for committing suicide). The last three levels of ideation were only evaluated if active thoughts of killing oneself were endorsed [27]. Current and past suicidal behavior was assessed through C-SSRS and supplemented by patient charts.

#### Suicide ideation

For screening purposes, suicide ideation was identified as a “yes” answer to the question, “Have you ever seriously thought about committing suicide?” (Kessler, Borges, & Walters, 1999). Ideation was corroborated by clinician report and quantified using the C-SSRS.

#### Suicide attempt

Suicide attempt is defined in the C-SSRS as a potentially self-injurious act committed with at least some wish to die as a result thereof. Suicide attempt is distinguished from non-suicidal self injurious behavior in the C-SSRS by questions assessing subjects intent to cause their own death versus to “relieve stress, feel better, get sympathy,” *et cetera*. [26].

#### Current attempt

Subjects were assessed for the presence of an actual suicide attempt at the time of presentation to the psychiatric emergency room by reconciliation of subject report in the C-SSRS and their history of present illness as recorded in their emergency room charts.

#### Actual attempt lethality

Actual suicide attempt lethality is rated in the C-SSRS on a 0–5 scale, from no or very minor injury requiring no care (0), to mild injury such as might be treated by first aid measures (1) to moderate, requiring some medical care (2), to moderate-severe injury, requiring hospitalization (3), to severe, requiring intensive care (4) to death (5). [26]. The actual lethality level of current attempts was assessed using the C-SSRS supplemented by patient charts.

#### Substantive current attempt

Subjects with a *Current Attempt* with *Actual Attempt Lethality* ≥2 were defined as having made a substantive current attempt.

### Statistical Method

#### Scale reliability

Scale reliability was assessed by calculation of Cronbach’s alpha, and individual scale items were assessed for their influence on Cronbach’s alpha.

#### Scale structure

As this is a novel scale, exploratory factor analysis was employed. However, as the scale was hypothesized to reflect an affective circumplex, predicting four latent factors – “positive self”, “negative self”, “positive other”, and “negative other”, a factor analytic approach using Maximum Likelihood extraction was used to test the hypothesized four-factor structure. Factor analysis was performed, and three- and four-factor models were compared, as only four factors had eigenvalues greater than one. Further, as depression is associated with negative biases [28], “negative self” and “negative other” factors should be expected to correlate. Thus non-orthogonal Oblimin factor rotation with Kaiser normalization was employed to derive an optimal factor structure. [29] Factors of the final model were then assessed as subscales using Cronbach’s alpha to measure subscale reliability. Subscales were generated by assigning each item loading above a threshold level of 0.3 [29] to the factor on which it loaded most strongly. In addition, as Cronbach’s alpha tends to be deflated for scales with fewer than seven items [30], mean inter-item correlation rho was calculated for each scale and compared to the reference ranges suggested by Clark and Watson. [31].

#### Convergent and divergent validity

Correlations of the AIRS total and subscale scores with subscales of the BSI were assessed using forward conditional regression of BSI subscales against each derived subscale of the AIRS.

All analyses were implemented in SPSS.

#### Construct validity and individual affect relations to suicide attempt

Separate binary logistic regression analyses individually regressing AIRS total score, AIRS subscale scores, and AIRS individual item scores against the dependent variable ‘current suicide attempt’ were performed. In secondary analyses these regressions were repeated using ‘substantive current attempt’ (i.e., attempts requiring at least some medical care) as the dependent variable. In addition, stepwise binary logistic regression was performed to establish the relations between actual suicide attempt (dependent variable) and demographic and diagnostic factors. Significant demographic and diagnostic factors were then added block-wise to the models testing association of AIRS scores with current attempt: stepwise binary logistic regression was performed for each of three models to establish the relations between current suicide attempt (dependent variable) and 1) AIRS total score, 2) AIRS subscale scores, and 3) AIRS individual item scores. In secondary analyses, interaction effects among significant AIRS items were examined, and a composite suicide-related subscale scale was generated and its properties as a predictor of suicide attempt status were tested using Receiver-Operator Characteristic analysis.

## Results

### Description of Sample

Demographic characteristics of the study sample are presented in Table 1. Our sample was approximately two-thirds male, and overwhelmingly single. Approximately two-thirds had annual income <$20,000, and were unemployed or disabled and had independent housing. Approximately half had no more high-school education.

**Table 1:** Demographic Characteristics of Participants.

Variable	Mean	SD	Variable		n	%
Age (range 21–65)	40.3	10.8	*Sex*			
			Male	112		64.2%
			Female	63		35.8%
			Male to Female transsexual	1		0.6%
**Variable**	**n**	**%**	**Variable**	**n**		**%**
*Income*			*Type of Housing*			
< $20,000	112	63.6%	In apartment, house or student housing		113	64.2%
$20,000–$39,999	25	14.2%	In psychiatric group home or residence		11	6.3%
$40,000–$59,999	12	6.8%	Homeless		34	19.3%
$60,000–$79,999	6	3.4%	Missing		18	10.2%
$80,000–$99,999	2	1.1%				
$100,000+	4	2.3%				
Missing	15	8.5%				
*Marital Status*			*Employment Status*			
Married	12	6.8%	Full-time		21	11.9%
Unmarried (total)	155	88.1%	Other		145	82.3%
Single/Never Married	98	55.7%	Unemployed		86	58.9%
Separated	22	12.5%	Part-time		13	8.9%
Divorced	26	14.8%	Disabled		38	26%
Widowed	9	5.1%	Volunteer		4	2.7%
Missing	9	5.1%	Retired		1	0.7%
			Household full-time		3	2.1%
			Missing		10	5.8%
*Years of Education*						
0–4 years	6	3.4%				
5–8 years	9	5.1%				
9–12 years	72	40.9%				
13–16 years	55	31.3%				
>16 years	11	6.3%				

Clinical characteristics of our sample are presented in Tables 2 and 3. Out of all subjects, 31 (17.6%) presented with actual suicide attempt and 145 (82.4%) reported only suicidal ideation. Of the 31 subjects with actual suicide attempt, 11 (35%) had a C-SSRS lethality level of 0 (no harm), 9 (29%) had a lethality level of 1 (minor harm), 7 (23%) had a lethality level of 2 moderate harm (requiring some medical care), 3 (10%) had a lethality level of 3 (requiring medical hospitalization), and 1 (3%) had a lethality level of 4 (requiring ICU care). Of the non-attempters, approximately two-thirds were male while among subjects presenting with current attempt the majority was female. The majority, 99 (56.3%) reported previous suicide behavior. The majority of both actual suicide attempters and subjects presently currently with only suicidal ideation reported a lifetime history of attempt. However, a substantial portion (12/31 = 38.7%) of attempters reported no past history of suicide attempt. From the entire sample, 92 (52.3%) participants reported alcohol use, drug use or both. More than half (90, 51.1%) of subjects were diagnosed with unipolar depression and/or anxiety disorder.

### Structure and Performance of the AIRS Measure

#### Reliability

The AIRS as a whole demonstrated good reliability with a Cronbach’s alpha of 0.747 (n = 172). No items increased alpha when removed from the scale.

#### Factor structure of the AIRS measure

Exploratory factor analysis identified four factors with eigenvalues greater than 1 and examination of the scree-plot suggested a 3 or 4 factor solution. Factor analysis using maximum likelihood factor extraction with Oblimin rotation was used to compare three-, and four-factor solutions. Only the four-factor solution provided a significant fit for the data with chi-square  = 94.26 (df 74, p = 0.056), while the three-factor solution did not (p-value of chi-square statistic <0.01). As predicted, Factor 1 (Negative Other) correlated most strongly with factor 3 (Negative Self), with r = 0.35. Other pairwise correlations between factors were modest, ranging from 0.03 to 0.13.

#### Subscales identified within the AIRS measure

Each item loading above 0.35 on a factor was assigned to the factor on which it loaded most strongly to generate subscales corresponding to each of the four factors. This yielded four subscales within the AIRS measure: ‘*Positive Self’, ‘Negative Self’, ‘Negative Other’, and ‘Jealousy’*. All items except ‘positive feelings towards other’ were assignable to a subscale. Cronbach’s alphas revealed good internal consistency for the first three subscales ranging from 0.771–0.668, with scale-length invariant rhos ranging from 0.29–0.36 suggesting good reliability for these broad constructs [31]. The Jealousy subscale consists of a single item, and thus cannot be analyzed for reliability. Factor and subscale structure and reliability statistics are detailed in Table 4.

**Table 2:** Characteristics of Suicide Attempters and Non-Attempters (N = 176).

	n (%)	Age Mean		Age SD
*Presented with Suicide Attempt*	31 (17.6%)	38.39		14.27
First attempt	12 (38.7%)			
Second attempt	6 (19.4%)			
Third attempt	5 (16.1%)			
Four attempt	1 (3.2%)			
Fifth attempt	4 (12.9%)			
Sixth attempt	3 (9.7%)			
* Presented with Suicide Ideation only*	145 (82.4%)	40.65	9.85	
With past attempt	99 (68.3%)			
No past attempt	46 (31.7%)			

**Table 3:** Distribution of Suicide Attempt and Ideation by Sex and Diagnostic cluster.

Presenting Level of Suicidality:			Axis I Diagnostic Category:				Total n
			Psychotic d/o	Bipolar, no psychosis	Unipolar + Anxiety d/o	No Axis I Dx	
SI only	Sex	male	22	8	54	16	100
		female	9	11	19	6	45
	Total n		31	19	73	22	145
Actual SA	Sex	male	1	2	9	1	13
		female	3	2	8	5	18
	Total n		4	4	17	6	31

**Table 4:** Factor Analysis & Resulting Subscales.

AIRS individual Items	Factor				Subscale:
	1	2	3	4	Cronbach’s alpha (rho)
Angry	**0.777**	0.053	0.152	−0.035	Negative Other Subscale:
Hatred	**0.679**	0.026	0.316	0.285	0.686 (0.356)
Disgust	**0.570**	0.077	0.520	−0.181	
Negative Other	**0.426**	0.133	0.148	0.206	
Joy	−0.002	**0.754**	0.045	0.087	Positive Self Subscale:
Positive self	0.139	**0.647**	0.141	0.044	0.771 (0.362)
Love	0.032	**0.627**	0.018	0.083	
Pride	0.165	**0.565**	0.068	0.112	
Calm	0.002	**0.534**	0.000	0.090	
Sexual desire	0.115	**0.484**	0.123	0.019	
Fear	0.116	0.188	**0.657**	−0.101	Negative Self Subscale:
Shame	0.297	0.208	**0.601**	0.145	0.709 (0.290)
Anxiety	0.224	0.030	**0.562**	0.007	
Negative self	0.229	−0.184	**0.549**	0.423	
Sad	0.326	−0.032	**0.395**	−0.255	
Jealousy	0.273	0.262	0.083	**0.441**	Jealousy Subscale: not applicable
Positive Other	0.260	0.211	0.187	0.179	Not Loading on Any Factor

Extraction Method: Maximum Likelihood. Rotation Method: Oblimin with Kaiser Normalization.

#### Convergent and discriminant validity

The AIRS subscales demonstrated good convergent and discriminant validity with subscales of the BSI. In forward conditional regression, the AIRS Positive Self subscale associated uniquely with psychotic and somatory subscales of the BSI (Standardized Beta  = 0.391 (p = 0.001), 0.175 (p = 0.033, respectively) and demonstrated a unique negative association with BSI depression (Standardized Beta −0.517, p<0.0005). The AIRS Negative Self subscale demonstrated unique positive associations with BSI depression (Standardized Beta 0.370, p<0.0005) and interpersonal problem subscales (Standardized Beta 0.242, p = 0.001), while the AIRS Negative Other subscale demonstrated unique positive association with BSI hostility (Standardized Beta 0.535, p<0.0005). Finally, Jealousy demonstrated a unique positive association with BSI hostility (Standardized Beta 0.197, p<0.021).

### Construct Validity and Relation to Suicidality

#### AIRS score distributions

In this sample of subjects presenting to the ER with suicidality, total scores on the AIRS ranged from 0 to 33 out of a possible 34 points and averaged 16.9 points, demonstrating wide variability. Scores were only slightly skewed towards higher score (skewness −0.10). However, mean score on the Positive Self was low (3.4, range 0–12) and the distribution was more substantially skewed to lower scores (skewness  = 0.64). Scores were highest for Negative Self (mean 7.6, range 0–10), and their distribution demonstrated strong skewness towards higher scores (−0.89). Scores were also only somewhat skewed towards higher score for the Negative Other subscale (mean 4.7, range 0–8, skewness −0.22). Scores for Jealousy were low (mean 0.56, range 0–2), and strongly skewed towards low score (skewness 0.96).

#### Association of AIRS total and subscale scores with suicide attempt status

Significant clinical and demographic factors were identified for inclusion in the model should significant bivariate relations be found for scale total, subscale, and individual item scores. Gender, age, marital status, years of education, annual income, and presence or absence of substance abuse were entered into a stepwise backwards logistic regression against attempt status. Of these, only two associated significantly with attempt status; presence of substance abuse (Beta −1.49, p = 0.004) and male gender (Beta −0.95, p = 0.036) had significant negative association with presence of an actual suicide attempt. Psychotic disorders as a group were also strongly negatively associated with actual SA (Beta −2.19, p = 0.023).

Regression of SA status against AIRS total score revealed no significant association, with or without control for gender, substance abuse, and diagnostic category. Regression of SA status against AIRS subscale scores also revealed no significant association, with or without control for gender, substance abuse, and diagnosis. These findings were unchanged when analysis was restricted to substantive suicide attempts.

### Individual Affect Relations with Suicide Attempt Status

One-at-a-time logistic regression of individual AIRS items against suicide attempt status as the dependent variable (without control for other variables), found only ‘Love’ a significant predictor of presentation with an actual attempt (Beta 0.915, p<0.0005) ‘Positive feelings towards self’ approached significance as a negative predictor (Beta −0.570, p = 0.061). For substantive suicide attempts only ‘Love’ remained a significant predictor (Beta 0.904, p = 0.03).

To account for item covariation, stepwise backwards conditional logistic regression of SA status against AIRS individual items was employed, and revealed three items which associated significantly with attempt status: a positive association for intense feelings of love (Beta  = 1.462, p<0.0005), negative association for feelings of calm (Beta  = −1.144, p = 0.012), and negative association for positive feelings directed towards oneself (Beta  = −0.984, p = 0.011) with suicide attempt. Addition of demographic and diagnostic factors to the regression did not significantly alter these results, and among the added variables only negative association with the presence of substance abuse remained significant (Beta  = −5.91, p = 0.003). Thus, the presence of love and absence of calm or positive feelings towards oneself and substance abuse were significant factors for actual suicide attempt. In repetition of this analysis with outcome restricted to substantive attempts only “Love” (Beta  = 1.537, p = 0.003) and “Calm” (Beta  = −2.737, p = 0.049) remained significant predictors.

Backwards conditional stepwise regression of significant AIRS Items, demographic factors (Gender and substance use), and their pairwise interactions revealed that while AIRS love remained a positive correlate of current attempt, the interaction of AIRS love with AIRS Calm was protective (Beta = −1.48, p = 0.01). Further, interaction analysis of different levels of affects revealed that experiencing a lot of feelings of love simultaneously with no feelings of calm associated with approximately 13-fold increased odds of actual suicide attempt (Beta  = 2.58, p = 0.023). In repetition of this analysis with outcome restricted to substantive attempts the interaction was similar but not statistically significant.

**Figure 1:**
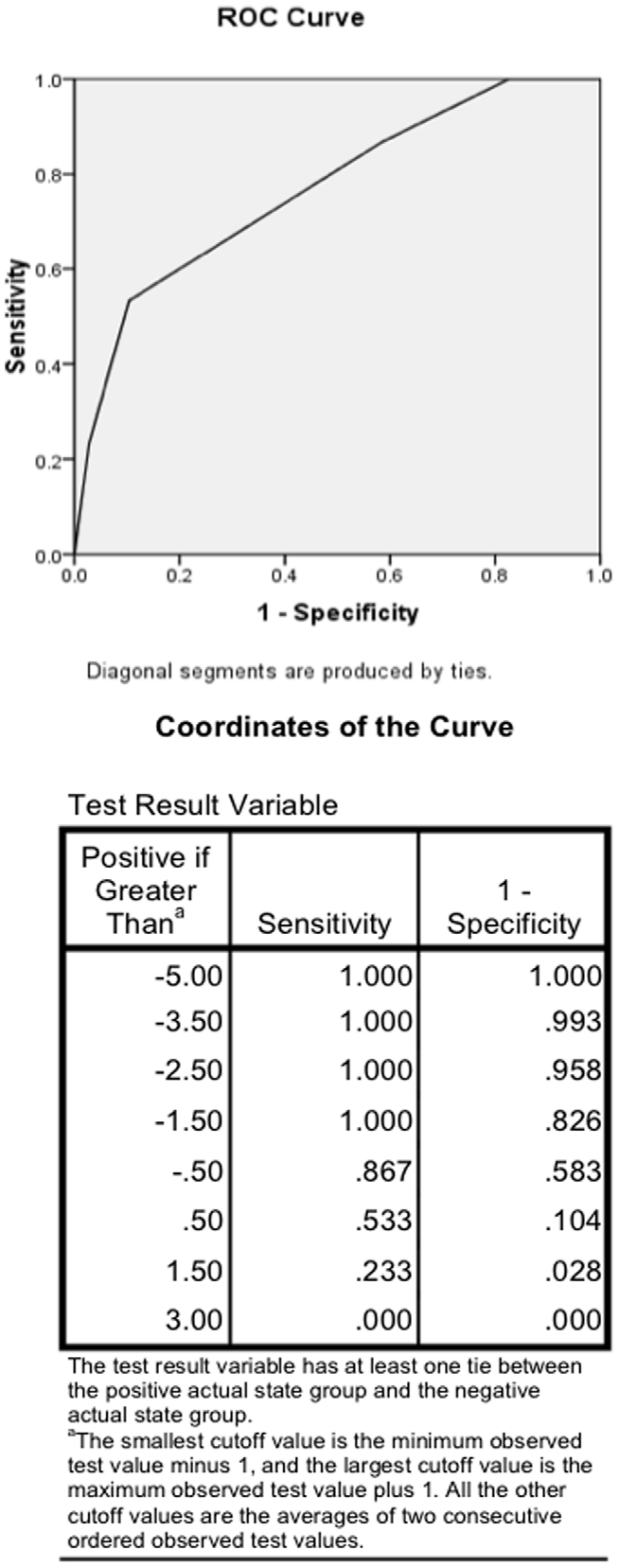
Receiver Operator Characteristic curve for the AIRS composite suicide risk score. This post hoc analysis shows the Receiver Operator Characteristic curve and its coordinates for the AIRS composite suicide risk score derived by subtracting scores on the individual items “Positive feelings towards self” and “Calm” from the individual item “Love”.

In a secondary analysis, a composite variable was created by subtracting scores on the Calm and Positive Self items from score on the Love item. Score on this composite variable significantly discriminated between subjects who presented with actual suicide attempt and those who presented with ideation only. Area under the curve for the Receiver Operator characteristic function was 0.768 (95% confidence interval 0.673–0.864, p<0.0005). At an optimal cut score of ≥0, sensitivity was 86.7% and specificity was 41.7%. A score of 1 or greater was 90% specific for presentation with actual suicide attempt, but had sensitivity of only 47%. (See figure 1.) In repetition of this analysis with outcome restricted to substantive attempts, performance was virtually unchanged with area under the curve 0.744 (p = 0.010), and optimal cut-score 0, yielding sensitivity 90% and specificity 38.4%.

## Discussion

In this study, we examined the structure of a novel scale, the 17-item Affective Intensity Rating Scale (AIRS), and its relation to suicidality in a psychiatric emergency room setting. While this is, first of all, a novel scale validation study, it also serves as the first study, to our knowledge, to systematically survey the affective experience of persons presenting to a psychiatric emergency room with acute suicidality. As such, we hypothesized that:

1. The AIRS would function as a reliable measure with a factor structure consistent with a circumplex model of affect comprising valence and directedness components, thus generating “Positive Self”, “Negative Self”, “Positive Other”, and “Negative Other” subscales.
2. Greater affective intensity as measured by AIRS total score would associate with subjects’ presenting to the ER with current attempt, versus ideation only.
3. High intensity self-directed negative affects (“negative self”) will associate with current attempt, versus ideation only.
4. The AIRS would identify individual affects linked to current attempt, versus ideation only.

Our first hypothesis was supported by the finding that the AIRS, despite comprising a diverse range of affects, functioned as an internally consistent scale with Cronbach’s alpha of 0.747 and all items contributing to the measure. Given that many individual items were nominally opposed (e.g., ‘love’ and ‘hate’) this result suggests that the scale total score is a reflection of a common factor of affective intensity. Our first hypothesis was also largely supported by the finding of three factors which generated reliable subscales: “Positive Self”, “Negative Self”, and “Negative Other”. Contrary to our expectation, however, “positive feelings towards others” did not emerge as factor, rather the individual item “Jealousy” emerged as a fourth factor separate from Negative Other. It is possible that positive other-directed feelings were underrepresented in the AIRS. Finally, jealousy may simply not fit into the proposed affective circumplex, being rather than a purely self- or other-directed affect, a complex composite. [32].

Furthermore, the AIRS subscales demonstrated good convergent and discriminant validity with subscales of the BSI. In regression analysis the BSI Hostility subscale was the sole subscale to associate significantly with AIRS Negative Other, while BSI Interpersonal difficulty and Depression subscales were the sole subscales to associate significantly with AIRS Negative Self. The strongest association between BSI subscales and AIRS Positive Self was, intuitively, an inverse relation with BSI Depression. Less intuitively, additional associations were also found between AIRS Positive Self and the BSI Psychotic and, more weakly, BSI Somatic subscales. One possible explanation of these modest associations is that disorganized affect in psychotic subjects accounts for the association between positive affects and psychotic symptoms, or that the immediate presence of caring personnel and raters elicits more positive affect in subjects with schizophrenia [33], [34].

Our second and third hypotheses were not supported in this study. However, as might be expected in a sample of suicidal subjects, score distributions are skewed towards high negative affect scores and low positive affect scores. It is worth noting however that a substantial percentage of subjects presenting to the psychiatric emergency room with suicidality also report recent experiences of intense positive affects. The role these experiences might have in resilience for such patients is an area worthy of further investigation [35], [36].

Our final hypothesis – that individual affects and specific affect combinations would associate with suicide attempt – was supported. This finding is in keeping with the notion that, given the absolute infrequency of suicide among individuals at risk [37], suicide must be driven not by the general depressive sector of the affect spectrum but by specific individual affects or affect combinations, as is suggested analogously, for example, in the ‘escape theory’ of suicide [38]. Thus, perhaps the most striking finding is the strong association between subjects endorsing strong feelings of love with current attempt status. Indeed, the role of intense feelings of love in suicide attempt might be understood in the context of attachment, given that insecure attachment is associated with higher risk for suicide attempt [39], and has been found to have significant interactions with depression at the level of brain activity [40], [41].

Furthermore, we found that feelings of calm moderated this association such that strong feelings of calm were protective while their absence increased risk of being a current suicide attempter among subjects reporting strong feelings of love. Finally the specific combination of intense feelings of love with low feeling of calm and low positive feeling towards self distinguished current suicide attempters from subjects who presented solely with suicide ideation. Though this result might appear surprising, a survey of suicide notes found that emotions of love, gratitude and forgiveness were expressed in most [42] **.** Further, in a study of 100 consecutive high lethality suicide attempters Hall et al., [19] found that after depressed mood the two most common features were severe anxiety (92%) and/or panic attacks (80%) along with recent loss of a close personal relationship (78%). These recurrent findings might be explained on the one hand, by the tremendous capacity of love to elicit distress, and on the other, by the notion that beyond the presence of action-promoting risk factors motivating suicidal behavior, it is the absence of action-inhibiting protective factors (such as, e.g., successful recruitment of internalized secure attachment representations and/or actual attachment figures) that allows the suicidal impulse to be acted upon. As distress is a key activator of the attachment system [43] distress triggered by loss of or rejection by an attachment object – a loved one – may, particularly in the context of insecure attachment, result in a suicide-promoting positive feedback [44] between distress and attachment system activation. Furthermore, as Coyne and colleagues have demonstrated such positive feedback my be reified in depression, as depressed subjects often elicit not only depression but hostility in others, including care-givers [45]–[47]. Such an interpretation is consistent with a previous study by Fisher et al., [48] which concluded that romantic rejection can produce feelings of immense loss and can lead to clinical depression and suicide. Persons beset by intense affective states with high distress-reactivity resulting in loss of emotional control are likely to act in ways that appear impulsive [49], including low-plan suicide attempts [50]. Indeed, in accord with a distinctive role for the absence of inhibiting protective factors in distinguishing suicide attempters from non-attempters, a key factor in distinguishing attempters appears to be reactivity to individual distress and/or showing more aggressive/impulsive tendencies [50], [51].

### Limitations

This study has several important limitations.

In terms of scale validation, the sample consisted only of English speaking psychiatric emergency room patients presenting with acute suicidality, which may limit generalizability. Further, convergent validity with other measures of affective intensity remains to be demonstrated.

In terms of clinical significance, the study is limited in that the sample is insufficiently powered to detect what might be real but small effects in the relationship between total and subscale scores and suicide attempt status. Moreover, this study captured only non-fatal, primarily low-lethality suicide attempts and potential participants who successfully attempted were perforce not included, and those who had made near lethal attempts were seen only after medical stabilization, and may have been less willing to participate in the study. Lastly, while endorsement of intense feelings of love associated significantly with current suicide attempt, as with any self-report on a descriptor, the interpretation of the descriptor is ultimately the subject’s. Thus the type of love affect experienced by attempters was not assessed, and the self-report of intense feelings of love could indicate romantic love, philial love, romantic rejection, loss of a loved one or some other form of love. While this study does not provide answers to these questions, our strong findings point to the importance of detailed investigation of the role of love and other attachment-related affects in suicide, and an examination of the balance between action-promoting risk factors, and action-inhibiting protective factors in suicidality.

### Conclusions

The AIRS is an instrument with three reliable subscales distinguishing positive and negative affect domains and self- vs. other- directedness of affects consistent with a tri-axial circumplex model of affect, as well as indication of jealousy as an independent fourth factor. Though affective intensity *per se* does not seem to predict suicidal behavior, intense feelings of love, particularly in the absence of protective feelings of calm or positive self view associated significantly with suicidal action. Thus, although this instrument focuses on an intrapersonal variable of state affective intensity, its primary clinical function appears to be the offer of a perspective on a person in an interpersonal context, using affective ratings to gain insights into the complex relational situations leading to a suicide attempt. Thus, our study points to the need for further investigation of affective intensity, love, and attachment-related relational dynamics in the evolution of suicidality.

## Supporting Information

Appendix S1 **This file shows the AIRS in its entirety.**
(DOCX)

## References

[1] National Center for Injury Prevention and Control CfDCaPA, (2008)GA: Centers for Disease Control and Prevention WISQARS fatal injuries: mortality reports. In: Centers for Disease Control and Prevention. Atlanta G, editor.

[2] National Center for Injury Prevention and Control CfDCaPA (2008)GA: Centers for Disease Control and Prevention WISQARS nonfatal injuries: nonfatal injury reports. In: Prevention CfDCa, editor. Atlanta, GA.

[3] Simon RI (2006) Imminent Suicide: The Illusion of Short-Term Prediction. Suicide and Life-Threatening Behavior 36: 296–301. 16805657 10.1521/suli.2006.36.3.296

[4] Hendin H, Maltsberger JT, Szanto K (2007) The Role of Intense Affective States in Signaling a Suicide Crisis. The Journal of Nervous and Mental Disease 195: 363–368 310.1097/NMD.1090b1013e318052264d. 10.1097/NMD.0b013e318052264d 17502800

[5] Hendin H, Al Jurdi RK, Houck PR, Hughes S, Turner JB (2010) Role of Intense Affects in Predicting Short-term Risk for Suicidal Behavior: A Prospective Study. The Journal of Nervous and Mental Disease 198: 220–225 210.1097/NMD.1090b1013e3181d1013d1014. 10.1097/NMD.0b013e3181d13d14 20216000

[6] Hendin H, Maltsberger JT, Haas AP, Szanto K, Rabinowicz H (2004) Desperation and Other Affective States in Suicidal Patients. Suicide and Life-Threatening Behavior 34: 386–394. 15585460 10.1521/suli.34.4.386.53734

[7] Yaseen Z, Katz C, Johnson M, Eisenberg D, Cohen L, et al (2010) Construct development: The Suicide Trigger Scale (STS-2), a measure of a hypothesized suicide trigger state. BMC Psychiatry 10: 110. 21144063 10.1186/1471-244X-10-110 PMC3016314

[8] Posner J, Russell JA, Peterson BS (2005) The circumplex model of affect: An integrative approach to affective neuroscience, cognitive development, and psychopathology. Development and Psychopathology 17: 715–734. 16262989 10.1017/S0954579405050340 PMC2367156

[9] Russell JA (1980) A circumplex model of affect. Journal of Personality and Social Psychology 39: 1161–1178. 10.1037//0022-3514.79.2.286 10948981

[10] Cacioppo JT, Petty RE, Losch ME, Kim HS (1986) Electromyographic activity over facial muscle regions can differentiate the valence and intensity of affective reactions. Journal of Personality and Social Psychology 50: 260–268. 3701577 10.1037//0022-3514.50.2.260

[11] Bachorowski J-A, Braaten EB (1994) Emotional intensity: Measurement and theoretical implications. Personality and Individual Differences 17: 191–199.

[12] Larsen RJ, Diener E (1985) A multitrait-multimethod examination of affect structure: hedonic level and emotional intensity. Personality and Individual Differences 6: 631–636.

[13] Larsen RJ, Diener E (1987) Affect intensity as an individual difference characteristic: A review. Journal of Research in Personality 21: 1–39.

[14] Flett GL, Hewitt PL (1995) Criterion validity and psychometric properties of the affect intensity measure in a psychiatric sample. Personality and Individual Differences 19: 585–591.

[15] Weiner B (2000) Intrapersonal and Interpersonal Theories of Motivation from an Attributional Perspective. Educational Psychology Review 12: 1–14.

[16] Bolton JM, Pagura J, Enns MW, Grant B, Sareen J (2010) A population-based longitudinal study of risk factors for suicide attempts in major depressive disorder. Journal of Psychiatric Research 44: 817–826. 20122697 10.1016/j.jpsychires.2010.01.003 PMC2888712

[17] Hirsch JK, Duberstein PR, Chapman B, Lyness JM (2007) Positive affect and suicide ideation in older adult primary care patients. Psychology and Aging 22: 6. 10.1037/0882-7974.22.2.380 PMC4846281 17563193

[18] Canetto SS, Lester D (2002) Love and achievement motives in women’s and men’s suicide notes. The Journal of psychology 136: 573–576. 12431040 10.1080/00223980209605552

[19] Hall RCW, Platt DE, Hall RCW (1999) Suicide Risk Assessment: A Review of Risk Factors for Suicide in 100 Patients Who Made Severe Suicide Attempts: Evaluation of Suicide Risk in a Time of Managed Care. Psychosomatics 40: 18–27. 9989117 10.1016/S0033-3182(99)71267-3

[20] Chen Y-W, Dilsaver SC (1996) Lifetime rates of suicide attempts among subjects with bipolar and unipolar disorders relative to subjects with other axis I disorders. Biological Psychiatry 39: 896–899. 8860192 10.1016/0006-3223(95)00295-2

[21] Kessler RC, Borges G, Walters EE (1999) Prevalence of and Risk Factors for Lifetime Suicide Attempts in the National Comorbidity Survey. Arch Gen Psychiatry 56: 617–626. 10401507 10.1001/archpsyc.56.7.617

[22] Lieberman PB, Baker FM (1985) The reliability of psychiatric diagnosis in the emergency room. Hospital & community psychiatry 36: 291–293. 3979981 10.1176/ps.36.3.291

[23] Warner MD, Peabody CA (1995) Reliability of diagnoses made by psychiatric residents in a general emergency department. Psychiatric services 46: 1284–1286. 8590116 10.1176/ps.46.12.1284

[24] Cheniaux E, Landeira-Fernandez J, Versiani M (2009) The diagnoses of schizophrenia, schizoaffective disorder, bipolar disorder and unipolar depression: interrater reliability and congruence between DSM-IV and ICD-10. Psychopathology 42: 293–298. 19609099 10.1159/000228838

[25] Derogatis LR, Melisaratos N (1983) The Brief Symptom Inventory: an introductory report. Psychological Medicine 13: 595–605. 6622612

[26] Posner K, Brown GK, Stanley B, Brent DA, Yershova KV, et al (2011) The Columbia–Suicide Severity Rating Scale: Initial Validity and Internal Consistency Findings From Three Multisite Studies With Adolescents and Adults. The American Journal of Psychiatry 168: 1266–1277. 22193671 10.1176/appi.ajp.2011.10111704 PMC3893686

[27] Mundt JC, Greist JH, Gelenberg AJ, Katzelnick DJ, Jefferson JW, et al (2010) Feasibility and validation of a computer-automated Columbia-Suicide severity rating scale using interactive voice response technology. Journal of Psychiatric Research 44: 1224–1228. 20553851 10.1016/j.jpsychires.2010.04.025

[28] Rude SS, Wenzlaff RM, Gibbs B, Vane J, Whitney T (2002) Negative processing biases predict subsequent depressive symptoms. Cognition & Emotion 16: 423–440.

[29] Floyd FJW, Keith F (1995) Factor analysis in the development and refinement of clinical assessment instruments. Psychological Assessment 7: 286–299.

[30] Spiliotopoulou G (2009) Reliability reconsidered: Cronbach’s alpha and paediatric assessment in occupational therapy. Aust Occup Ther J 56: 150–155. 20854508 10.1111/j.1440-1630.2009.00785.x

[31] Clark LA, Watson D (1995) Constructing validity: Basic issues in objective scale development. Psychological Assessment 7: 309–319. 10.1037/pas0000626 PMC6754793 30896212

[32] Buunk BP, Angleitner A, Oubaid V, Buss DM (1996) Sex Differences in Jealousy in Evolutionary and Cultural Perspective: Tests From the Netherlands, Germany, and the United States. Psychological Science 7: 359–363.

[33] Aleman A, Kahn RS (2005) Strange feelings: Do amygdala abnormalities dysregulate the emotional brain in schizophrenia? Progress in Neurobiology 77: 283–298. 16352388 10.1016/j.pneurobio.2005.11.005

[34] Kring AM, Moran EK (2008) Emotional Response Deficits in Schizophrenia: Insights From Affective Science. Schizophrenia Bulletin 34: 819–834. 18579556 10.1093/schbul/sbn071 PMC2632476

[35] Siegel K, Meyer IH (1999) Hope and Resilience in Suicide Ideation and Behavior of Gay and Bisexual Men Following Notification of HIV Infection. AIDS education and prevention : an interdisciplinary journal : official publication of the International Society for AIDS Education 11: 53–64. 10070589

[36] Meadows LA, Kaslow NJ, Thompson MP, Jurkovic GJ (2005) Protective Factors Against Suicide Attempt Risk Among African American Women Experiencing Intimate Partner Violence. American Journal of Community Psychology 36: 109–121. 16134048 10.1007/s10464-005-6236-3

[37] Holma KM, Melartin TK, Haukka J, Holma IAK, Sokero TP, et al (2010) Incidence and Predictors of Suicide Attempts in DSM–IV Major Depressive Disorder: A Five-Year Prospective Study. American Journal of Psychiatry 167: 801–808. 20478879 10.1176/appi.ajp.2010.09050627

[38] Chatard A, Selimbegovic L (2011) When Self-Destructive Thoughts Flash Through the Mind: 39. Failure to Meet Standards Affects the Accessibility of Suicide-Related Thoughts. Journal of Personality and Social Psychology 100: 587–605. 21299310 10.1037/a0022461

[39] Lizardi D, Grunebaum MF, Burke A, Stanley B, Mann JJ, et al (2011) The effect of social adjustment and attachment style on suicidal behaviour. Acta Psychiatr Scand 124: 295–300. 21644941 10.1111/j.1600-0447.2011.01724.x PMC3785084

[40] Galynker, II, Yaseen ZS, Katz C, Zhang X, Jennings-Donovan G, et al. (2011) Distinct but overlapping neural networks subserve depression and insecure attachment. Soc Cogn Affect Neurosci. 10.1093/scan/nsr074 PMC3501706 22037687

[41] Zhang X, Yaseen ZS, Galynker, II, Hirsch J, Winston A (2011) Can depression be diagnosed by response to mother’s face? A personalized attachment-based paradigm for diagnostic fMRI. PLoS ONE 6: e27253. 22180777 10.1371/journal.pone.0027253 PMC3236742

[42] Sanger S, Veach PM (2008) The interpersonal nature of suicide: a qualitative investigation of suicide notes. Arch Suicide Res 12: 352–365. 18828039 10.1080/13811110802325232

[43] Mikulincer M, Gillath O, Shaver PR (2002) Activation of the attachment system in adulthood: threat-related primes increase the accessibility of mental representations of attachment figures. J Pers Soc Psychol 83: 881–895. 12374442

[44] Katz C, Yaseen ZS, Mojtabai R, Cohen LJ, Galynker II (2011) Panic as an independent risk factor for suicide attempt in depressive illness: findings from the National Epidemiological Survey on Alcohol and Related Conditions (NESARC). J Clin Psychiatry 72: 1628–1635. 21457675 10.4088/JCP.10m06186blu

[45] Coyne JC (1976) Depression and the response of others. Journal of abnormal psychology 85: 186–193. 1254779 10.1037//0021-843x.85.2.186

[46] Strack S, Coyne JC (1983) Social confirmation of dysphoria: shared and private reactions to depression. J Pers Soc Psychol 44: 798–806. 6842366 10.1037//0022-3514.44.4.798

[47] Kahn J, Coyne JC, Margolin G (1985) Depression and Marital Disagreement: The Social Construction of Despair. Journal of Social and Personal Relationships 2: 447–461.

[48] Fisher HE, Brown LL, Aron A, Strong G, Mashek D (2010) Reward, Addiction, and Emotion Regulation Systems Associated With Rejection in Love. Journal of Neurophysiology 104: 51–60. 20445032 10.1152/jn.00784.2009

[49] Mann JJ, Waternaux C, Haas GL, Malone KM (1999) Toward a clinical model of suicidal behavior in psychiatric patients. Am J Psychiatry 156: 181–189. 9989552 10.1176/ajp.156.2.181

[50] Oquendo MA, Galfalvy H, Russo S, Ellis SP, Grunebaum MF, et al (2004) Prospective study of clinical predictors of suicidal acts after a major depressive episode in patients with major depressive disorder or bipolar disorder. Am J Psychiatry 161: 1433–1441. 15285970 10.1176/appi.ajp.161.8.1433

[51] Zouk H, Tousignant M, Seguin M, Lesage A, Turecki G (2006) Characterization of impulsivity in suicide completers: Clinical, behavioral and psychosocial dimensions. Journal of affective disorders 92: 195–204. 16545465 10.1016/j.jad.2006.01.016

