# Supplementary material for: Love and Suicide: The Structure of the Affective Intensity Rating Scale (AIRS) and Its Relation to Suicidal Behavior
Source: PLoS One. 2012 Aug 29;7(8):e44069. doi: 10.1371/journal.pone.0044069 (PMC3430651; doi:10.1371/journal.pone.0044069)
Supplement: Appendix S1 — This file shows the AIRS in its entirety. (DOCX) [file pone.0044069.s001.docx]

# Appendix 1.

### AIRS version 1:

I would like to ask you some questions about the way you were feeling over the last several days when you were dealing with the thoughts, emotions, and events that resulted in you coming to the emergency room feeling that you might kill yourself or want to kill yourself.

During the last 3 days before you came to the Emergency Room did you experience any periods where you felt the following:

1. Unusually intense or deep feelings of sadness?

0=Not at all

1=Sometimes

2=A lot

1. Unusually intense or deep feelings of anger?

0=Not at all

1=Sometimes

2=A lot

1. Unusually intense or deep feelings of disgust?

0=Not at all

1=Sometimes

2=A lot

1. Unusually intense or deep feelings of sexual desire?

0=Not at all

1=Sometimes

2=A lot

1. Unusually intense or deep feelings of hatred?

0=Not at all

1=Sometimes

2=A lot

1. Unusually intense or deep feelings of calm?

0=Not at all

1=Sometimes

2=A lot

1. Unusually intense or deep feelings of shame?

0=Not at all

1=Sometimes

2=A lot

1. Unusually intense or deep feelings of love?

0=Not at all

1=Sometimes

2=A lot

1. Unusually intense or deep feelings of jealousy?

0=Not at all

1=Sometimes

2=A lot

1. Unusually intense or deep feelings of joy?

0=Not at all

1=Sometimes

2=A lot

1. Unusually intense or deep feelings of fear?

0=Not at all

1=Sometimes

2=A lot

1. Unusually intense or deep feelings of pride?

0=Not at all

1=Sometimes

2=A lot

1. Unusually intense or deep feelings of anxiety?

0=Not at all

1=Sometimes

2=A lot

1. Any unusually intense or deep positive feelings directed towards yourself?

0=Not at all

1=Sometimes

2=A lot

1. Any unusually intense or deep positive feelings directed towards someone else?

0=Not at all

1=Sometimes

2=A lot

1. Any unusually intense or deep negative feelings directed towards yourself?

0=Not at all

1=Sometimes

2=A lot

1. Any unusually intense or deep negative feelings directed towards someone else?

0=Not at all

1=Sometimes

2=A lot
